# Clinical and polysomnographic predictors of laryngopharyngeal reflux in obstructive sleep apnea syndrome^☆^

**DOI:** 10.1016/j.bjorl.2018.03.007

**Published:** 2018-04-14

**Authors:** Fábio Azevedo Caparroz, Milena de Almeida Torres Campanholo, Caroline Gomez Regina, Sung Woo Park, Leonardo Haddad, Luís Carlos Gregório, Fernanda Louise Martinho Haddad

**Affiliations:** a Universidade Federal de São Paulo (UNIFESP), Escola Paulista de Medicina (EPM), *Fellowship* em Rinologia, São Paulo, SP, Brazil; b Associação Médica Brasileira (AMB), Certificação em Medicina do Sono, São Paulo, SP, Brazil; c Universidade Federal de São Paulo (UNIFESP), Escola Paulista de Medicina (EPM), Programa de Pós-Graduação em Medicina em Otorrinolaringologia, São Paulo, SP, Brazil; d Universidade Federal de São Paulo (UNIFESP), Escola Paulista de Medicina (EPM), Departamento de Otorrinolaringologia e Cirurgia de Cabeça e Pescoço – Setor de Rinologia, São Paulo, SP, Brazil; e Universidade Federal de São Paulo (UNIFESP), Escola Paulista de Medicina (EPM), Medicina, São Paulo, SP, Brazil; f Universidade Federal de São Paulo (UNIFESP), Escola Paulista de Medicina (EPM), Ambulatório de Laringologia, São Paulo, SP, Brazil; g Universidade Federal de São Paulo (UNIFESP), Escola Paulista de Medicina (EPM), Ambulatório de Disfagia do Setor de Laringe e Voz, São Paulo, SP, Brazil; h Universidade Federal de São Paulo (UNIFESP), Escola Paulista de Medicina (EPM), Medicina em Otorrinolaringologia, São Paulo, SP, Brazil; i Universidade Federal de São Paulo (UNIFESP), Escola Paulista de Medicina (EPM), Departamento de Otorrinolaringologia e Cirurgia de Cabeça e Pescoço – Setor de Laringe, São Paulo, SP, Brazil; j Associação Brasileira de Otorrinolaringologia e Cirurgia Cérvico-Facial (ABORLCCF), Departamento de Medicina do Sono, São Paulo, SP, Brazil; k Universidade Federal de São Paulo (UNIFESP), Escola Paulista de Medicina (EPM), Departamento de Otorrinolaringologia e Cirurgia de Cabeça e Pescoço – Ambulatório de Distúrbios do Sono, São Paulo, SP, Brazil

**Keywords:** Obstructive sleep apnea, Gastroesophageal reflux, Laryngopharyngeal reflux, Apneia obstrutiva do sono, Refluxo gastroesofágico, Refluxo laringofaríngeo

## Abstract

**Introduction:**

Obstructive sleep apnea syndrome and laryngopharyngeal reflux are diseases with a high prevalence in the overall population; however, it remains unclear whether they are diseases with the same risk factors present in the same populations or if there is any association between them.

**Objectives:**

To evaluate and determine the prevalence of laryngopharyngeal reflux in patients with moderate and severe obstructive apnea syndrome and also to determine its predictive factors.

**Methods:**

Historical cohort, cross-sectional study of patients aged 18–70 years, referred to a tertiary service Otorhinolaryngology outpatient clinic with a polysomnographic diagnosis of moderate or severe obstructive sleep apnea syndrome. The reflux symptom index questionnaire and the reflux finding score at indirect videolaryngoscopy were applied to the assessed population, considering the inclusion and exclusion criteria.

**Results:**

Fifty-six patients were evaluated, of which 64.3% had a positive laryngopharyngeal reflux (positive reflux symptom index and/or positive endolaryngeal reflux finding score). Body mass index was a predictor of reflux presence in this group of patients with moderate to severe obstructive sleep apnea syndrome. In patients with positive score for endoscopic findings and reflux symptom index (12.3%), there was a trend toward significance for a higher mean apnea–hypopnea index and a higher percentage of sleep time with oxyhemoglobin saturation below 90% (*p* = 0.05).

**Conclusion:**

The prevalence of laryngopharyngeal reflux was higher in this group of patients with moderate to severe obstructive sleep apnea syndrome, and the body mass index was a predictor of laryngopharyngeal reflux in these patients. There was a trend toward greater oxyhemoglobin desaturation in patients with a positive score for reflux symptoms index (RSI) and reflux finding score (RFS).

## Resumo

**Introdução:**

A síndrome da apneia obstrutiva do sono e o refluxo laringofaríngeo são doenças com alta prevalência na população em geral. No entanto, ainda não está claro se são doenças com os mesmos fatores de risco presentes nas mesmas populações ou se há alguma relação entre elas.

**Objetivo:**

Avaliar e determinar a prevalência de refluxo laringofaríngeo em pacientes com síndrome da apneia obstrutiva moderada e acentuada, bem como determinar os fatores preditivos de refluxo nesses pacientes.

**Método:**

Estudo de coorte histórica com corte transversal de pacientes entre 18 e 70 anos, encaminhados a um ambulatório de Otorrinolaringologia em serviço terciário, com diagnóstico polissonográfico de síndrome da apneia obstrutiva do sono moderada ou acentuada. Foram aplicados o questionário *Reflux Sympton Index* e o escore de achados endolaríngeos por meio de videolaringoscopia indireta na população estudada, respeitando os critérios de inclusão e exclusão.

**Resultados:**

Foram avaliados 56 pacientes, dos quais 64,3% apresentaram refluxo laringofaríngeo (*Reflux Sympton Index* positivo e/ou *Reflux Sympton Index* positivo). O índice de massa corpórea foi fator preditor da presença de refluxo laringofaríngeo nesse grupo de pacientes com síndrome da apneia obstrutiva do sono moderada e acentuada. Nos pacientes cujos *Reflux Sympton Index* e o escore de achados endolaríngeos foram positivos (12,3%), houve uma tendência à significância para maior índice de apneia e hipopneia e maior porcentagem do tempo de sono com saturação de oxi-hemoglobina abaixo de 90% (*p* = 0,05).

**Conclusão:**

A prevalência de refluxo laringofaríngeo foi alta nesse grupo de pacientes com síndrome da apneia obstrutiva do sono moderada e acentuada e o índice de massa corpórea foi fator preditor de refluxo nesses pacientes. Houve uma tendência a maior dessaturacão de oxi-hemoglobina em pacientes com “índice de sintomas de refluxo” e escore de achados endolaríngeos positivos.

## Introduction

Obstructive sleep apnea syndrome (OSAS) and laryngopharyngeal reflux (LPR) are conditions with high prevalence in the general population, affecting 2–4% and 20–40% of the adult population, respectively.^1^, ^2^ Both have a significant impact on quality of life and lead to increased morbidity in affected individuals.^1^, ^2^ However, although their association has been suggested in the literature, it is unclear whether this association actually exists or whether they are conditions with the same risk factors, coexisting in the same populations.^3^, ^4^, ^5^

There are several theories supporting the association between laryngopharyngeal reflux and OSAS, with the main ones based on inflammation and decrease in the upper airway caliber due to prolonged contact of the gastroduodenal content with the pharynx and larynx.^4^ Moreover, both diseases share obesity as an important risk factor, which may justify apnea and reflux in the same individual due to the reduction in upper airway permeability and increasing intra-abdominal pressure.^4^

It is known that sleep decreases the effectiveness of the upper esophageal sphincter (UES) when acting as a barrier against laryngopharyngeal reflux, particularly during slow-wave sleep. UES pressure also varies with breathing, with minimal values during expiration. The pressure of the lower esophageal sphincter (LES), on the other hand, is also minimal during expiration, but is not affected by the sleep stages.^6^

In a recent study carried out in Brazil, the prevalence of signs and symptoms suggestive of laryngopharyngeal reflux in adults with OSAS was 89%, significantly higher in obese individuals.^7^ However, population studies evaluating this correlation and prevalence between the diseases are still lacking.

The aim of the present study is to evaluate and determine the prevalence of laryngopharyngeal reflux (LPR) in patients diagnosed with moderate and severe obstructive sleep apnea syndrome (OSAS) and also to determine its predictive factors.

## Methods

This was a clinical cross-sectional study of a historical cohort of patients aged 18 to 70 years, referred to a tertiary service Otorhinolaryngology outpatient clinic with a polysomnographic diagnosis of moderate to severe obstructive sleep apnea syndrome (OSAS), according to the criteria of the last International Classification of Sleep Disorders (2014). The patients came to the outpatient clinic with a primary complaint of snoring and/or excessive daytime sleepiness (EDS), and after a clinical and polysomnographic diagnosis of moderate or severe OSAS, they underwent evaluation and screening for laryngopharyngeal reflux (LPR), using standardized scores of signs and symptoms of the latter condition.

The patients were submitted to all-night polysomnography. The polygraph used was EMBLA^®^ S7000 (Embla Systems, Inc., Broomfield, CO, USA), with monitoring of electroencephalogram, electromyogram (mentonian region and anterior tibial muscles), electrocardiogram, airflow (nasal cannula associated with pressure transducer and oronasal thermistor), respiratory effort (thorax and abdomen), peripheral oxyhemoglobin (SpO_2_) saturation, snoring and body position. Sleep staging was performed according to the criteria proposed by Rechtschaffen and Kales^8^ and awakenings according to the criteria of the American Sleep Disorders Association (ASDA).^9^ The analysis of respiratory events was performed according to the criteria proposed by the American Academy of Sleep Medicine (AASM).^10^

Patients using proton pump inhibitors (PPIs) or other gastric medications in the past 30 days, patients submitted to previous esophageal surgery, those with decompensated clinical and/or psychiatric diseases, users of alcohol or other psychoactive substances, users of sedative medications (that could alter esophageal tone) and patients with neuromuscular diseases were excluded. Patients who were already using continuous positive airway pressure (CPAP) therapy for the last 3 months were also excluded.

The study was submitted to and approved by the Institutional Ethics and Research Committee/Platforma Brasil under number 195196 (CAAE 35375814.0.0000.5505).

Study participation was voluntary. The patients who agreed to participate in the study signed the Free and Informed Consent Form (FICF), previously submitted to and approved by the Institutional Ethics and Research Committee.

**Figure 1:**
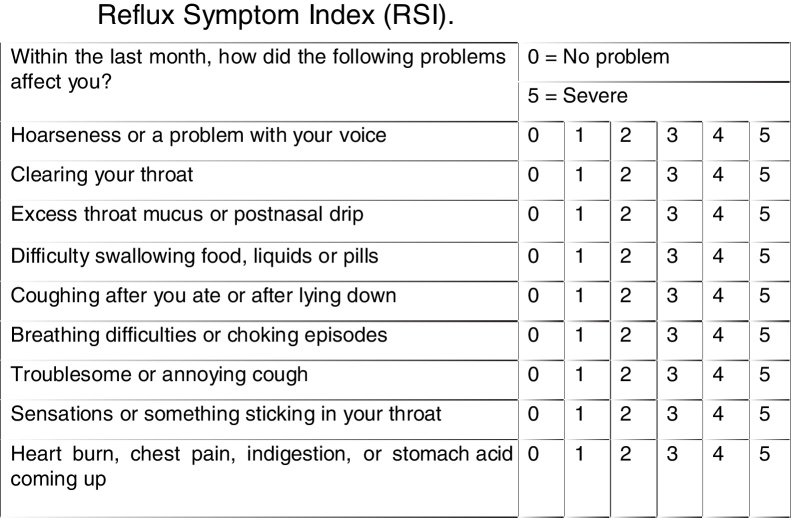
Reflux symptom index (RSI).

**Figure 2:**
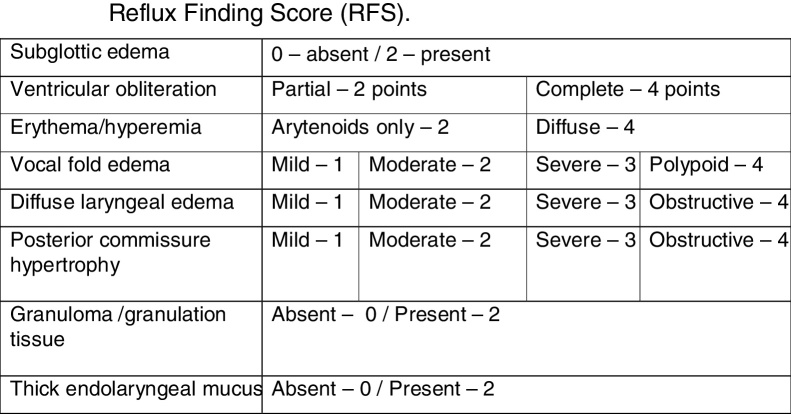
Reflux finding score (RFS).

The questionnaire on laryngopharyngeal reflux symptoms – the reflux symptom index (RSI) (Fig. 1) and the Endolaryngeal Reflux Findings Scale (EAER) or reflux finding score (RFS) (Fig. 2) were always applied by the same examiner after indirect laryngoscopy performed with a Machida 3.2 mm flexible nasofibroscope. Aiming to reduce the subjectivity of the Endolaryngeal Reflux Findings Scale assessment (EAER or RFS), the examination was always performed by two laryngologists, who jointly scored for each parameter considered in the RFS score. The maximum time between polysomnography and clinical evaluation with the use of the questionnaires and indirect laryngoscopy was 6 months.

Individuals who had a score >13 in the RSI questionnaire and/or a score >7 in the RFS scale, according to the original description of the scores, were considered as having laryngopharyngeal reflux.^11^, ^12^ The questionnaire and scale were translated, adapted and validated into Brazilian Portuguese^13^, ^14^, ^15^ and their final versions are shown in Figure 1, Figure 2.

### Statistical analysis

The statistical analysis was performed using the SPSS program (version 19.0 for Windows). The Kolmogorov–Smirnov test was used to verify the normality of the variables. Levenne's test was used to verify the homogeneity of the variables between the groups.

The descriptive analysis of the continuous variables was represented by means ± standard deviation, and the comparison of the variables was carried out by the general linear model (GLM) test for the normal data distribution. The Mann–Whitney test (*p* < 0.05) was performed for non-parametric data (*p* < 0.05).

Pearson's correlation was performed for the preliminary analyses. Logistic regression models were used for the analysis of reflux predictors (LPR) and the concomitant presence of RFS and RSI.

The categorical variables were represented by relative frequency (percentage) and the chi-square test was used to compare the groups. The significance level was set at *p* < 0.05.

## Results

A total of 59 patients were evaluated, of which 33.9% were females and 66.1% males. Three patients were excluded from the statistical analyses due to lack of data (incomplete records). The mean age of the evaluated patients was 49 ± 11 years and the mean body mass index (BMI) was 31.8 ± 4.7 kg/m^2^. The mean apnea–hypopnea index (AHI) was 52.0 ± 30.6 events/h, minimal oxyhemoglobin saturation was 75.2 ± 20.7%, and the mean percentage of total sleep time with oxyhemoglobin saturation <90% was 73 ± 9.7%. Considering as a criterion of positivity for laryngopharyngeal reflux, the score >13 in the RSI tool or score >7 in the RFS, according to the classification of the original study descriptions, we observed that 64.3% of the patients can be considered as having laryngopharyngeal reflux (positive RSI or RFS). However, when we considered positive RSI and RFS for this diagnosis, we found a significantly lower percentage of the sample, 12.5%.

It was verified that the percentage of patients with positive RSI was 37.9%, with the mean score being 13.7 ± 9; the percentage of patients with positive RFS was 38.6%, and the mean score was 6.9 ± 3.9.

No statistically significant differences were found in the clinical and polysomnographic parameters assessed in the groups of patients who scored positively and negatively for laryngopharyngeal reflux in the RSI and RSF questionnaires, when assessed alone.

When comparing the groups of patients with and without laryngopharyngeal reflux according to the criterion of positive RSI or RFS, it was observed that BMI was higher with statistical significance (*p* = 0.03) in the group of patients with reflux (Table 1) and was also higher with statistical significance in the group of patients who scored positively in both tools (*p* = 0.02) (Table 2).

**Table 1:** Descriptive data of the sample according to the LPR classification (positive RSI or RFS).

Numerical variables	LPR− (*n* = 20)	LPR+ (*n* = 36)	*p*	Effect size	Observed power
	Mean ± SD	Mean ± SD			
Age (years)	50.0 ± 14.2	48.7 ± 10.5	0.70	0.003	0.06
BMI (kg/m^2^)	30 ± 3.2	32.8 ± 5.3	0.03^a^	0.08	0.57
NC (cm)	41.6 ± 4.1	41.5 ± 9.9	0.97	0.00	0.05
AHI	27.6 ± 13.3	37.9 ± 32.9	0.19	0.03	0.24
AHI/h	41.9 ± 20.7	57.5 ± 34.9	0.07	0.05	0.43
Sat. O_2_ min. (%)	75.2 ± 7.4	72.1 ± 10.6	0.27	0.02	0.19
% Sat < 90%	15.5 ± 16.9	20.9 ± 22.1	0.38	0.01	0.13

LPR, laryngopharyngeal reflux; RSI, reflux symptom index; RFS, reflux finding score; BMI, body mass index; NC, neck circumference; AHI, arousal index per hour of sleep; AHI/h, apnea–hypopnea index per hour of sleep; RDR, respiratory disturbance rate per hour of sleep; Sat. O_2_ min., minimal oxyhemoglobin saturation; % Sat < 90%, oxyhemoglobin saturation percentage below 90%; *p*, level of statistical significance.

a Test (general linear model), *p* < 0.05.

**Table 2:** Sample descriptive data according to the RSI and RFS positive classification.

Numerical variables	Others (*n* = 49)	RSI and RFS+ (*n* = 7)	*p*	Size effect	Observed power
	Mean ± SD	Mean ± SD			
Age (years)	50.0 ± 12.0	43.3 ± 8.4	0.16	0.03	0.27
BMI (kg/m^2^)	31.8 ± 4.5	31.3 ± 7.1	0.74	0.02^a^	0.05
NC (cm)	41.5 ± 4.5	41.5 ± 6.6	0.87	0.00	0.05
AHI	30.8 ± 22.5	58.0 ± 48.4	0.14	0.10	0.69
AHI/h	48.6 ± 27.9	75.0 ± 44.8	0.05^a^	0.07	0.56
Sat. O_2_ min. (%)	74.2 ± 9	66.8 ± 11.8	0.10	0.01	0.17
% Sat < 90%	17.3 ± 18.9	40.9 ± 30.5	0.05^a^	0.06	0.48

LPR, laryngo-pharyngeal reflux; RSI, reflux symptom index; RFS, reflux finding score; BMI, body mass index; NC, neck circumference; AHI, arousal index per hour of sleep; AHI/h, apnea–hypopnea index per hour of sleep; RDR, respiratory disturbance rate per hour of sleep; Sat. O_2_ min., minimal oxyhemoglobin saturation; % Sat < 90%, oxyhemoglobin saturation percentage below 90%; *p*, level of statistical significance.

a *p* < 0,05.

In the group of patients who had positive RSI and RFS (12.5%), concomitantly, there was a trend toward significance (*p* = 0.05 – Mann–Whitney test) for higher apnea and hypopnea index and higher percentage of sleep time with oxyhemoglobin saturation <90% (Table 3). There were no differences regarding gender when comparing the groups.

**Table 3:** Predictors for LPR (positive RSI or RFS).

	*B*	*p*	Exp (*B*)	95%CI	
				Lower limit	Upper limit
Age (years)	0.027	0.457	1.027	0.957	1.102
BMI (kg/m^2^)	0.259	0.016^a^	1.296	1.050	1.599
Female gender	−0.714	0.438	0.490	0.080	2.979
AHI/h	0.036	0.071	1.037	0.997	1.079
Sat. O_2_ min. (%)	0.023	0.687	1.024	0.914	1.146
% Sat < 90%	−0.011	0.706	0.989	0.936	1.045

LPR, laryngopharyngeal reflux; RSI, reflux symptom index; RFS, reflux finding score; BMI, body mass index; NC, neck circumference; AHI/h, apnea–hypopnea index per hour of sleep; Sat. O_2_ min., minimal oxyhemoglobin saturation; %Sat < 90%, oxyhemoglobin saturation percentage below 90%; *p*, level of statistical significance; *B*, logistic regression coefficient; *p*, level of statistical significance; Exp (*B*), estimated odds ratio; 95% CI, 95% confidence interval.

a Logistic regression, *p* < 0.05.

The logistic regression showed that BMI was a predictor factor for the presence of laryngopharyngeal reflux (considering the positive RSI or RFS criterion) (Table 3) in patients with moderate to severe obstructive sleep apnea syndrome, with an increase in the risk having reflux being about 30% higher in patients with an increase in BMI (95% CI: 1.050–1.599). However, no predictors were identified when evaluating the presence of laryngopharyngeal reflux according to the criteria of both positive RSI and RFS tools (Table 4).

**Table 4:** Predictors for positive RFS and RSI.

	*B*	*p*	Exp (*B*)	95%CI	
				Lower limit	Upper limit
Age (years)	–	0.999	1.000	0.857	1.167
BMI (kg/m^2^)	0.139	0.442	1.149	0.806	1.637
Female gender	−1.549	0.427	0.212	0.005	9.726
AHI-h	0.016	0.645	1.016	0.950	1.087
Sat. O_2_ min. (%)	−0.041	0.706	0.960	0.774	1.189
% Sat < 90%	0.025	0.566	1.025	0.941	1.117

LPR, laryngopharyngeal reflux; RSI, reflux symptom index; RFS, reflux finding score; BMI, body mass index; NC, neck circumference; AHI-h, apnea–hypopnea index per hour of sleep; Sat. O_2_ min., minimal oxyhemoglobin saturation; %Sat < 90%, oxyhemoglobin saturation percentage below 90%; *p*, level of statistical significance; *B*, logistic regression coefficient; *p*, level of statistical significance; Exp (*B*), estimated odds ratio; 95% CI, 95% confidence interval.

## Discussion

Gastroesophageal reflux disease (GERD) is a high-prevalence condition in the overall population and defined as symptoms and/or complications caused by the return of gastric and/or duodenal contents to the esophagus and/or upper aerodigestive tract. Retrosternal pyrosis and acid regurgitation are the most characteristic symptoms of the disease and, once present, they can establish GERD diagnosis without the need for complementary tests. Among the extraesophageal symptoms of GERD are sleep disturbances, chest pain, coughing, throat clearing, asthma and dental erosions.^1^, ^3^

Jaspersen et al., in a study including 6215 patients with GERD, verified that extraesophageal manifestations were observed in 32.8% of them. In the same study, disease duration for a period of more than one year and smoking showed a statistically significant association with the presence of extraesophageal disease manifestations.^11^ In parallel, GERD has been frequently diagnosed in patients with OSAS.^16^

The term laryngopharyngeal reflux started being used after a Conference in New Orleans, Louisiana, USA, in September of 1995, by James Koufman et al.^17^ Otorhinolaryngologists, gastroenterologists and pulmonologists participated in the consensus. The consensus report was published in 1996, and established the initial differences between LPR and GER:

1. Regarding the symptoms: many patients with LPR do not have dyspepsia. Common symptoms include throat clearing, globus pharyngeus, halitosis, prolonged vocal warm-up time, frequent sore throats, chronic cough, asthma, among others;
2. Regarding symptom periodicity: patients with LPR have intermittent symptoms, which are often not daily;
3. Regarding the time of day and body position in which reflux episodes occur: patients with LPR have episodes of daytime reflux and in the upright position (“upright daytime refluxers”), whereas patients with GERD have reflux episodes during the night and in the supine position (“nocturnal supine refluxers”);^18^
4. In relation to gastrointestinal motility and the presence of esophageal lesions and/or alterations: in patients with LPR, there is no dysmotility and/or esophagitis;^18^
5. In relation to physiopathogeny: LPR is related to a dysfunction of the upper esophageal sphincter (UES), whereas the GER is related to the transient relaxation episodes of the lower esophageal sphincter (LES);^6^
6. Regarding treatment: LPR usually requires higher doses of proton-pump inhibitors (PPIs) to control symptoms in relation to GER.^17^

However, often both conditions may be associated in the same individual. LPR has been identified in approximately 50% of patients diagnosed with GERD.^19^ Some authors have included in the diagnosis of laryngopharyngeal reflux the presence of three or more pharyngeal reflux events with pH < 5, recorded by 24-h double-probe pH-monitoring.^19^ One of the problems associated with this diagnostic criterion is the fact that pH monitoring detects only the presence of acid reflux and does not detect the presence of alkaline reflux. In this sense, the use of combined impedance-pH monitoring has been recommended as a more accurate diagnostic method. However, even with this diagnostic method, it is not always during the monitoring period that the patient will have reflux episodes sufficient for the diagnosis, very often originally related to life habits that were not present during the examination.^20^

Even though the most recent studies show that there is no gold standard for the diagnosis of LPR or agreement between the diagnostic methods,^5^ we consider the fact that the patients in our study did not undergo combined impedance-pH monitoring for comparative purposes a limiting factor of the study.

Thus, a questionnaire known as the reflux symptom index (RSI) was developed in 2001 aimed to approach a more probable diagnosis of laryngopharyngeal reflux (Fig. 1). In the original questionnaire validation study, 25 patients with a diagnosis of laryngopharyngeal reflux by double-probe pH monitoring (with the proximal probe located 1 cm higher than the upper esophageal sphincter) were evaluated before treatment and 6 months after the use of Proton-Pump Inhibitors (PPIs) administered in two daily doses, and compared with a group of 25 individuals in the Control Group without the diagnosis of LPR. It was verified that the mean RSI score in the LPR group after 6 months of treatment was statistically significantly close the score of the Control Group, consisting of asymptomatic individuals. The mean RSI score in asymptomatic subjects was 11.6 (95% CI 9.7–13.6). This normality value was significantly lower than in individuals with LPR not yet submitted to treatment, but it was also statistically similar to the value observed in individuals with LPR submitted to 6 months of treatment with PPIs. Thus, it was concluded that an RSI score >13 was compatible with the diagnosis of LPR.^11^ Subsequently, the questionnaire was translated and culturally adapted to Brazilian Portuguese.^15^ In our study, the percentage of patients with positive RSI was 37.9%, with a mean score of 13.7 ± 9.

In 2001, another tool was also developed for the diagnosis of laryngopharyngeal reflux, a score based on the findings of indirect laryngoscopy, known as reflux finding score (RFS) (Fig. 2). In the original study, 40 patients diagnosed with LPR confirmed by double-probe pH monitoring were initially evaluated through RFS, as well as after 2, 4 and 6 months of treatment with PPIs, and compared with a control group of 40 asymptomatic subjects submitted to RFS.

The scores were calculated by two independent and blinded laryngologists in the original study. The mean RFS score for the individuals in the control group was 5.2 (95%CI 3.6–6.8). The conclusion of the study is that, for an individual with an RFS score >7, there is 95% certainty of the diagnosis of laryngopharyngeal reflux.^12^ Subsequently, the RLF was translated, adapted and validated into Brazilian Portuguese, receiving the name of Endolaryngeal Findings Reflux Score (EAER).^13^, ^14^ In our study, the percentage of patients with positive RFS was 38.6%, and the mean score was 6.9 ± 3.9. We considered the fact that the videolaryngoscopy analysis performed by the two independent laryngologists who were blinded or submitted to paired analysis a limiting factor of our study.

Because the RSI questionnaire was patient-dependent only, and also because the percentages found between positive RSI (37.9%) and positive RFS (38.6%) were very similar in our study, we believe these findings may be even closer to the reality in patients with OSAS, unlike other previous studies that showed higher percentages of these data.^7^ Furthermore, we decided to consider a positive diagnosis of laryngopharyngeal reflux with only one positive tool (RFS or RSI), as well as the results of the original validation studies for these tools by Belafsky et al., as described above.^11^, ^12^ Therefore, the prevalence of laryngopharyngeal reflux in our study was 64.3%.

Belafsky et al. demonstrated that the indirect LPR findings, including the RFS score, tend to show a slower improvement in relation to reflux symptoms in patients undergoing treatment with PPIs for a period of 6 months.^21^ This can be considered a potential bias of our study and others that take laryngeal findings into account, even though patients using PPIs were excluded from our study.

Regarding the association between LPR and GERD, it is known that once reflux has been diagnosed in symptomatic patients through the reflux finding score (RFS) and the reflux symptom index (RSI), less than 40% of these patients have the diagnosis of GERD confirmed by more specific methods, such as 24-h pH monitoring.^22^ Recent studies corroborate the fact that there is no gold standard for the diagnosis of LPR, since there is no agreement between diagnostic methods, even considering the most accurate ones.^5^, ^23^, ^24^ The presence of classic symptoms and signs of laryngopharyngeal reflux that respond to PPIs in many patients with normal combined impedance-pH monitoring confirms these findings.^23^, ^24^

In a recent systematic review, Karkos et al. concluded there is an association between laryngopharyngeal reflux and OSAS, but with no evidence of a direct causal association.^5^ There are still few well-designed and controlled studies in this regard. Several factors can generate bias in this analysis, since they are multifactorial diseases influenced by anatomical alterations, body mass and comorbidities.^25^

In a national study of 105 patients, an association was found between higher RSI scores and apnea severity in obese patients (BMI > 30 kg/m^2^). The mean values of RSI in the non-obese group (*n* = 66 patients) were similar for mild (RSI = 11.96) and moderate/severe OSAS (RSI = 11.43). As for the mean RSI values in the obese group (*n* = 39), they were statistically different between mild OSAS (RSI = 6.7) and moderate/severe OSAS (RSI = 11.53). The same association was not found for RFS scores in both obese and non-obese groups. The conclusion of this study was that obese patients with moderate/severe OSAS have more severe symptoms than non-obese patients. As for non-obese patients, LPR and OSAS are independent factors, given the similar RSI scores.^25^ Additionally, another study with 24-h double-probe pH monitoring in OSAS patients showed that both the number and duration of reflux episodes during sleep significantly correlated with apnea severity.^26^ These results are in agreement with the findings of our study.

Regarding the clinical and polysomnographic parameters evaluated in our study, it was observed that BMI was higher, with statistical significance, in the group of patients with laryngopharyngeal reflux, at the same time being a predictor of the presence of reflux in patients with obstructive sleep apnea, disclosing data similar to those found by Xavier et al.,^7^ who found that the prevalence of laryngopharyngeal reflux was significantly higher in obese patients (BMI > 30 kg/m^2^) and that the significant difference in inflammatory signs suggestive of reflux between obese and non-obese subjects suggests that obesity may interfere with inflammatory findings in the pharynx and larynx.

The apnea and hypopnea index (AHI) and percentage of time with oxyhemoglobin saturation <90% were not identified as predictors of reflux in the assessed patients, but they showed to be parameters with borderline significance (*p* = 0.05) in the comparison between the groups of patients who showed positive RSI and RFS. It is possible that patients with a positive score in both tools that assess reflux have more severe forms of laryngopharyngeal reflux. In these patients, a trend toward greater desaturation of oxyhemoglobin was observed, which suggests that more severe forms of reflux may be predisposed by a greater severity of OSAS.

## Conclusion

It can be concluded that the occurrence of laryngopharyngeal reflux in patients with OSAS was higher in comparison to previous population studies; and that a higher body mass index was a predictor of the presence of reflux in these patients. There was a trend toward greater oxyhemoglobin desaturation in patients with positive scores in both, reflux finding scores and reflux symptoms index tools.

## Conflicts of interest

The authors declare no conflicts of interest.
